# Effects of Oxidation Modification by Malondialdehyde on the Structure and Functional Properties of Walnut Protein

**DOI:** 10.3390/foods11162432

**Published:** 2022-08-12

**Authors:** Lingge Sun, Qingzhi Wu, Xiaoying Mao

**Affiliations:** School of Food Science and Technology, Shihezi University, Shihezi 832000, China

**Keywords:** MDA, protein oxidation, structure, functional properties

## Abstract

(1) Background: The effects of protein oxidization induced by malondialdehyde (MDA), which was selected as a representative of lipid peroxidation products, on the structure and functional properties of walnut protein were investigated. (2) Methods: Walnut protein isolate was produced by alkali-soluble acid precipitation. The modification of walnut protein isolate was conducted by MDA solutions (0, 0.01, 0.1, 1, and 10 Mm), which were incubated in the dark for 24 h. (3) Results: Increased carbonyl content and the degradation of sulfhydryl groups indicated MDA-induced protein oxidization. The circular dichroism spectra revealed disruption of the ordered protein secondary structure. The change in the tertiary conformation of the MDA-treated protein was observed through intrinsic fluorescence. Small polypeptide chain scission was observed at low MDA concentrations (≤0.1 mM) and protein aggregation was observed at high MDA concentrations (>0.1 mM) using high-performance size exclusion chromatography. Oxidized protein solubility was reduced. Furthermore, the emulsification stability index, foam capacity, and foam stability of walnut proteins were increased after treatment with 0.1 mM of MDA. An excessive concentration of MDA (>0.1 mM) decreased emulsification and foaming properties. (4) Conclusions: These results show that MDA oxidation modified the structure of walnut protein and further affected its function, which should be taken into account in processing walnut protein products.

## 1. Introduction

The walnut, a member of the walnut family, is one of the four most prominent nuts in the world. Its lipid, protein, and carbohydrate content is 64%, 18–24%, and 12–16%, respectively. Walnuts also contain high levels of vitamins, minerals, and polyphenols that are beneficial for human health. In recent years, walnuts have become increasingly popular and valued because of their high nutritional and economic interest. Walnut protein extracted from walnut meat has been widely used in the food processing industry because of its excellent functional properties [1]. The walnut protein complement is broken down as 70.1% glutelin, 17.6% globulin, 6.8% albumin, and 5.3% prolamin [2], and its amino acid composition meets human nutritional requirements. However, proteins are the major target of oxidation, and protein nutritional and functional properties can be affected by oxidation [3] during storage, preparation, processing, and manufacturing. Direct attacks from reactive oxygen species, and the subsequent interaction of the protein with secondary oxidation products, such as MDA, result in covalent structural modification of proteins [4]. Lipid oxidation can cause multiple structural changes to proteins, such as breakage of the polypeptide chain, oxidative modification of amino acid residue side chains, and crosslinking between protein molecules [5]. This may result in the loss of the quality and nutritional components of food [6]. While lipid oxidation has been studied in depth [7], the relationship between protein oxidation and lipid peroxidation is examined in more detail in the present study. Lipid peroxidation products, including free radicals, lipid hydroperoxides, and reactive aldehyde derivatives, are capable of protein modification. Ye [8] reported that oxidation by peroxyl radicals deteriorates the emulsion properties of peanut protein isolate. The results reported by Wu et al. [9] show that acrolein alters the structure of soy protein. The contribution of MDA to the modification of proteins both in food and in vivo has been demonstrated previously [10]. Free radicals and lipid hydroperoxides are unstable, but reactive aldehydes derived from lipid peroxidation are stable and show high reactivity and relative longevity in protein oxidation [11]. The functional properties of proteins, such as emulsification and foaming (both widely used in the food industry), are governed by intrinsic properties of protein conformation, including molecular structure and physicochemical characteristics [12]. The content of MDA can be used to characterize the degree of lipid oxidation and, as the most abundant reactive aldehyde in lipid peroxidation secondary products, MDA is the most abundant of the α, β-unsaturated aldehydes [10] and induces protein oxidation by reacting with an amine group of a protein, producing a Schiff base [13]. However, at present, no report has mentioned the effect of MDA oxidation on the structure and functional properties of walnut protein. To investigate the effects of MDA oxidization on the structure and functional properties of walnut protein, walnut proteins were incubated with different concentrations of MDA. This study provides reference information on walnut protein oxidation induced by lipid peroxidation.

## 2. Materials and Methods

### 2.1. Materials

Walnut was purchased from a local market in the city of Shihezi in XinJiang province, China. Analytical grade acrylamide, 1, 1, 3, 3-tetraethoxypropane; 2, 4-dinitrophenylhydrazine (DNPH); N, N-methylene-bis-acrylamide; and other chemicals and reagents were provided by Western Asia Chemical Industry Co. (Shandong, China).

### 2.2. Preparation of Walnut Protein Isolate

Walnut protein isolate was produced according to Mao [14]. The peeled walnut kernel was defatted with hexane and stirred for 2 h at ambient temperature, and then the solvent was removed by vacuum filtration. Defatted walnut flour was dissolved in 26-fold deionized water and adjusted to a pH of 11 with 1 M of NaOH. The resuspended walnut flour was stirred for 2 h at room temperature and then centrifuged at 8000 r/min for 30 min (Fermerfeld Technology Co., Shanghai, China). The supernatant pH was adjusted to 4.5 with 1 M of HCl and then centrifuged again, as described above. The pH of the precipitate after centrifugation was adjusted to neutral with 1 M of NaOH. Finally, the precipitate was freeze-dried to produce walnut protein isolate flour.

### 2.3. Modification of Walnut Protein with MDA

The MDA was prepared according to Adams [10], with slight modifications. A total of 1100 mg of 1, 1, 3, 3-tetraethoxypropane was dissolved in 50 mL of 0.1 M HCl and stirred for 30 min at room temperature. The solution was heated in a water bath for 60 min at 50 °C. The concentration of MDA was determined at 267 nm using a UV752 Spectrophotometer (Shanghai spectroscopy instrument Co., Shanghai, China). Oxidative walnut protein was prepared according to Wu [3]. The MDA was mixed with walnut protein flour in 10 mM of sodium phosphate buffer (pH 7.4) at concentrations of 0, 0.01, 0.1, 1, and 10 mM. The MDA/walnut protein solutions were incubated with continuous shaking at 25 °C in the dark for 24 h. Then, 72-h dialysis at 4 °C in deionized water was used to remove unreacted MDA, and the walnut protein solution was freeze-dried to produce an MDA-modified walnut protein isolate.

### 2.4. The Measurement of Carbonyl Content of Walnut Protein

Protein carbonyl was evaluated using DNPH according to Huang [15]. A 5 mg/mL walnut protein solution was prepared with deionized water, and the protein content was determined by the Biuret protein assay. The carbonyl content of walnut protein samples was expressed by the molar extinction coefficient of DNPH at 367 nm.

### 2.5. The Measurement of Free Sulfhydryl and Total Sulfhydryl Content

Free sulfhydryl groups and total sulfhydryl groups in the walnut protein isolate were evaluated according to Beveridge T. et al. [16]. The oxidized walnut protein samples were dissolved in deionized water, and the protein concentration was determined by the Biuret method. A total of 3 mL of protein solution, 0.01 mol/L pH 8.0 of phosphate buffer (containing 1 mmol/L EDTA and 1% SDS), and 0.1 mL of DNTB was evenly shaken and placed in a water bath at 25 °C for 1 h. This was then returned to room temperature and centrifuged for 20 min at 10,000× *g*. Then, the supernatant was used to measure the absorbance at a 412-nm wavelength, and the free sulfhydryl content was calculated. The control group did not add DNTB. A total of 1 mL of the protein solution, 0.05 mL of mercaptoethanol, and 4 mL of urea-guanidine hydrochloride mixed solution was added to a water bath at 25 °C for 1 h, followed by 10 mL of 12% trichloroacetic acid, and repeated at 25 °C for 1 h. The water bath was then removed for 1 h, followed by a return to room temperature and centrifuging at 5000× *g* for 10 min to obtain a precipitate. To remove the unreacted mercaptoethanol, 20 mL of 12% trichloroacetic acid was added and centrifuged twice. After centrifugation, 10 mL of 0.1 mol/L pH 8.0 phosphate buffer (containing 1 mmol/L EDTA and 1% SDS) and 0.08 mL of DNTB were added, shaken evenly, and centrifuged in a water bath at 25 °C for 1 h. Then, the supernatant was used to measure the absorbance at a 412-nm wavelength, and the total sulfhydryl content was calculated.

$$total\;sulhydryl\;content\left(nmol/mg\right)=70.67\times A\times \frac{P}{C}$$

where *P* is the dilution factor, *C* is the protein concentration in milligrams per milliliter, and A is the absorbance at 412 nm.

### 2.6. The Measurement of Circular Dichroism (CD) Spectra

The circular dichroism (CD) spectra of walnut protein samples were evaluated according to Wu [9], using a MOS-450 Circular dichroism spectrometer (Biologic, French). Solutions of the walnut protein samples (50 μL/mL) were prepared with deionized water. The CD data were expressed as mean molar ellipticity [q] (deg cm^2^ dmol^−1^).

### 2.7. Intrinsic Fluorescence

The intrinsic fluorescence of walnut proteins was evaluated according to Wu [9], using an F-7000 fluorescence spectrometer (Hitachi, Kyoto, Japan). A walnut protein solution of 20 mg/mL was prepared in sodium phosphate buffer (10 mM, pH 7.4), along with a sodium phosphate buffer blank. The excitation wavelength was 295 nm, and the emission wavelength ranged from 300 to 500 nm.

### 2.8. High-Performance Size Exclusion Chromatogram (HPSEC)

An LC-20A high-performance liquid chromatograph (Shimadzu, Japan) and a TSKgel SW G4000 SWXL column were used to determine the molecular weight distribution of walnut protein, according to Wu [17]. Walnut proteins (5 mg mL^−1^) were extracted in sodium phosphate buffer (0.05 M, pH 8.0) containing sodium chloride (0.3 M) for 4 h at 25 °C with constant magnetic stirring, then centrifuged at 10,000× *g* for 10 min at 25 °C. The supernatant was filtered through a cellulose acetate membrane with a pore size of 0.45 μm (Sartorius Co, Ltd., Gottingen, Germany). The flow rate was 1 mL min^−1^ using phosphate buffer (0.05 mol L^−1^, 0.3 mol L^−1^ NaCl, pH 7.0) as the mobile phase. About 10 μL of the protein solutions were injected into the columns, and the eluent was monitored at 280 nm. Nine standard proteins (thyroglobulin, aldolase, bovine serum albumin (BSA), ovalbumin, peroxidase, adenylate kinase, myoglobin, ribonuclease A, and aprotinin) were used to generate a calibration curve.

The solubility of walnut protein was evaluated according to Ye [18], with a slight modification. The walnut protein samples were dissolved in deionized water and centrifuged (8000 r/min, 10 min). The protein concentration was evaluated by the Biuret protein assay using BSA as a standard. The percentage of protein solubility was expressed as the ratio of protein content in the supernatant to the total protein content in the sample.

$$\mathrm{Solubility}\left(\%\right)=\frac{\mathrm{protein}\;\mathrm{content}\;\mathrm{in}\;\mathrm{supernatant}}{total\;\mathrm{protein}\;\mathrm{content}\;\mathrm{in}\;\mathrm{sample}\;}\times 100$$

### 2.9. Foam Capacity (FC) and Foam Stability (FS)

The foaming capacity (FC) and foam stability (FS) were determined according to Zhang [19]. A total of 100 mL of walnut protein solution was homogenized using a high-speed homogenizer (FA 25 model, ELE Equipment Co., Ltd., Shanghai, China) at 10.000 rpm/min for 2 min at room temperature and then left to stand for 30 min. The volumes of foam and solution after whipping and standing for 30 min were recorded. FC and FS were calculated using the following equations.

$$\mathrm{FC}\left(\%\right)=\frac{volume\;after\;whipping-volume\;before\;whipping}{volume\;before\;whipping}\times 100\;\mathrm{FS}\left(\%\right)=\frac{volume\;after\;standing-volume\;before\;whipping}{volume\;after\;whipping}\times 100$$

### 2.10. Emulsifying Properties

The emulsifying activity index (EAI) and emulsion stability index (ESI) of walnut protein were determined according to Pearce and Kinsella [20]. A total of 3 mL of soy oil was added to 9 mL of 1% walnut protein solution dissolved in deionized water. The mixture was homogenized for 2 min at 10 Kr/min and then let stand for 10 min. A total of 10 μL of emulsion was mixed in 10 mL of 0.1% SDS at 0 and 10 min. The absorbance of the emulsion at 0 and 10 min was measured at 500 nm using a UV752 Spectrophotometer. The EAI and ESI were calculated using the following equations. The determination of protein content was as described above.

$$\mathrm{EAI}\left(m^{2}/g\right)=\frac{2\times 2.303\times A_{0}\times N}{c\times \varphi \times 10000}$$

$$\mathrm{ESI}\left(\min\right)=\frac{A_{0}}{A_{0}-A_{10}}\times 10$$

where *A*_0_ and *A*_10_ are the absorbance of the emulsion sample at 0 and 10 min, respectively; *φ* is the oil fraction, 0.25; c is the content of protein; *N* is the dilution factor, 100.

### 2.11. Statistical Analysis

All the experiment values were repeated three times and reported as mean ± SD. The statistical analysis was performed by a one-way ANOVA in SPSS 23.0 (SPSS, Inc). A significance analysis (*p* < 0.05) was performed by Duncan’s multiple range test.

## 3. Results

### 3.1. Characterization of the Oxidation of Walnut Protein

Protein carbonylation produced by the oxidation of amino acid side chains and peptide bonds is a common result of oxidative stress-induced protein modification [21], and the protein carbonyl content is an important characteristic of protein oxidation. The effects of oxidation modification by MDA on the carbonyl content of walnut protein are listed in Table 1. The results show that the carbonyl content of walnut protein without MDA was 3.12 nmole/mg. However, the carbonyl content of walnut protein after incubation with 10 mM of MDA was seven times higher than that of walnut protein without MDA. The results indicate that MDA causes a significant concentration-dependent increase in protein carbonyl formation (*p* < 0.05). Wu [3] reported that the carbonyl content of soybean protein increases gradually with the increase in MDA concentration. MDA reacts with beramine in protein molecules to form an enamine adduct, which is used to introduce a carbonyl group to proteins [22]. In addition, MDA can result in an increase in carbonyl by forming a Schiff base with the nucleophilic side chains of cystine, histidine, and lysine residues of proteins [23].

The effects of MDA oxidation on the free and total sulfhydryl content of walnut protein are listed in Table 1. Sulfhydryl group levels are another important parameter for estimating protein oxidation on cysteine residues, which cannot be characterized by the carbonyl content. The free and total SH content of untreated samples was 34.84 nmol/mg and 167.49 nmol/mg, respectively. With an increasing MDA concentration from 0 mM to 10 mM, there was a significant decrease in the free and total SH content (*p* 0.05). Meanwhile, the decrease in the total sulfhydryl group was greater than that of the free sulfhydryl group. This may be attributed to the conversion of sulfhydryl groups of cysteine residues into other sulfur-containing oxidized species besides disulfide bonds during incubation with MDA [9]. This phenomenon was also reported in Wang’s [24] study, in which about 30% of SH was lost after myofibrillar protein was incubated with 10 mM of MDA. Zhang and Lu [25] noted that disulfide bonds are associated with the stability of the emulsion and the protein’s molecular weight. Alleoni [26] indicated that the change in protein molecules caused by the disulfide bond can improve the formation of foam. The results above indicate that the oxidation process causes a sulfhydryl-disulfide exchange reaction between disulfide groups, which would alter the structural and functional properties of walnut proteins.

### 3.2. Circular Dichroism (CD) Spectra Measurement

**Figure 1:**
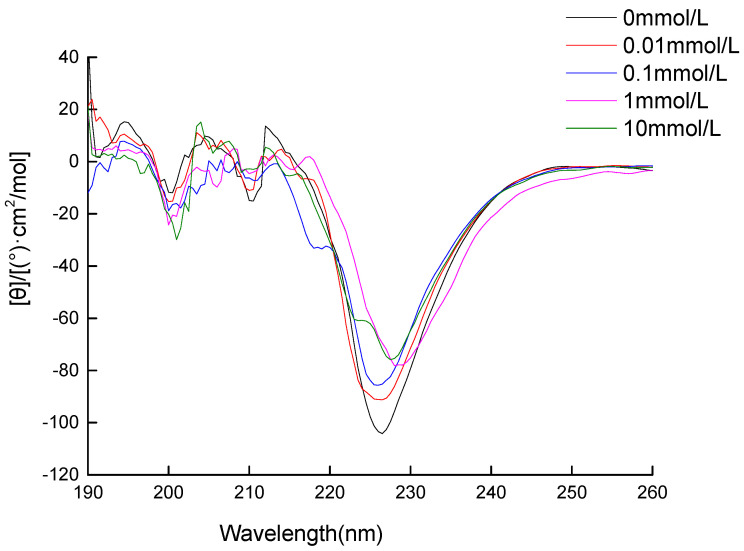
Circular dichroism spectra of walnut protein incubated with different concentrations of MDA (0 mM; 0.01 mM; 0.1 mM; 1 mM; and 10 mM).

Circular dichroism (CD) spectra detect conformational variations and the change in the protein secondary structure during oxidation. Figure 1 shows that the CD spectra of walnut protein without MDA exhibited two negative troughs at 210 nm and 226 nm, resulting from an α -helical structure that exhibits a negative Cotton effect; the positive peak at 194 nm indicates the existence of an β-sheet structure, and the negative troughs at 200 nm are characteristic of a disordered protein structure. When the MDA concentration was increased from 0 mM to 10 mM, the two negative bands at 210 nm and 226 nm and the positive peak at 194 nm showed a downward trend, which indicates that the oxidation caused the protein to lose its ordered secondary structure, specifically the α-helix and β-sheet. The negative band at 220 nm increased, which suggests that the random coil content also increased. Sun et al. [27] also found that a decrease in the percentage of α-helix conformations was related to protein oxidation. The CD spectra results indicate that the original stable secondary structure of walnut protein was destroyed by oxidation by MDA. The results show that the free sulfhydryl groups and total sulfhydryl groups decreased with the increase in MDA oxidation concentration, and indicate the structural changes of oxidized proteins, which to some extent affected the functional properties of oxidized walnut proteins.

### 3.3. Intrinsic Fluorescence

**Figure 2:**
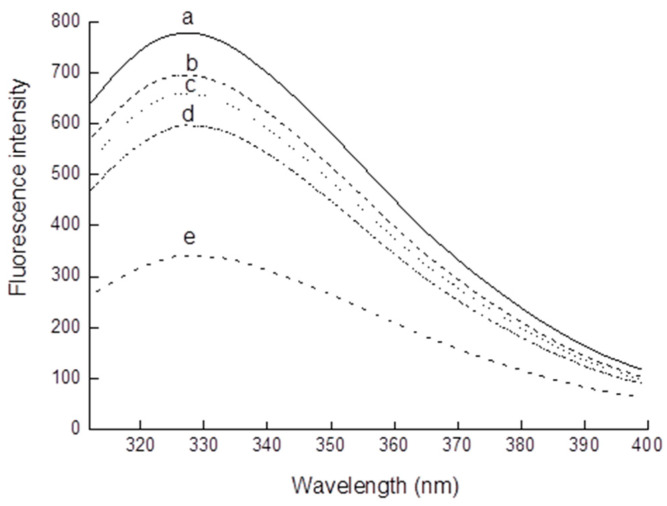
Intrinsic fluorescence of walnut protein incubated with different concentrations of MDA (a-0 mM; b-0.01 mM; c-0.1 mM; d-1 mM; and e-10 mM).

Intrinsic protein fluorescence mainly stems from tryptophan, a residue that is particularly sensitive to the polarity of its microenvironment. Intrinsic fluorescence spectroscopy is mostly used to reflect a tertiary protein structure by probing the hydrophilic or hydrophobic environment of tryptophan. Tryptophan residues can emit fluorescence in the range of 300–400 nm when excited at 290 nm [28]. As depicted in Figure 2, upon excitation at 295 nm, native walnut protein showed a maximal tryptophan fluorescence intensity at 328 nm. With increasing the concentration of MDA from 0 to 10 mM, the maximum emission wavelength of walnut protein blue-shifted slightly from 328 to 326 nm and the maximum fluorescence intensity decreased. This result is consistent with results reported by Wang [24]. This blue-shift of the maximum emission wavelength indicates that the exposed tryptophan residues were buried in the interior and led to a decrease in tryptophan fluorescence. King and Li [29] reported that fewer residues of tryptophan exposed in the aqueous environment caused by the coagulation of protein and specific modification of tryptophan residues were responsible for the decrease in tryptophan fluorescence. These results show that the tertiary structure of walnut protein was changed by MDA modification. Therefore, the intrinsic fluorescence and CD spectra results reveal that the advanced structure of walnut protein was destroyed by oxidation by MDA.

### 3.4. High-Performance Size Exclusion Chromatogram (HPSEC)

**Figure 3:**
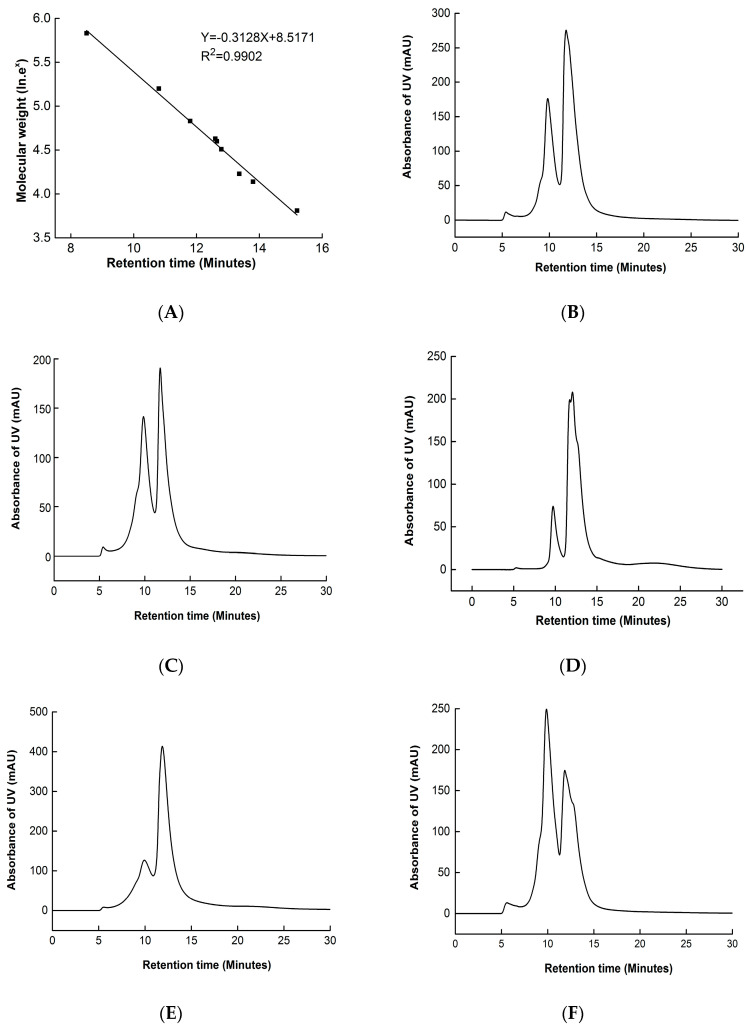
HPSEC of walnut protein incubated with different concentrations of MDA. Calibration curve of nine standard proteins used for interpreting the results: thyroglobulin (MW: 669,000); aldolase (MW: 158,000); BSA (MW: 67,000); ovalbumin (MW: 43,000); peroxidase (MW: 40,200); adenylate kinase (MW: 32,000); myoglobin (MW: 17,000); and ribonuclease. (**A**) (MW: 13,700), and aprotinin (MW: 6500); (**B**) 0 mM; (**C**) 0.01 mM; (**D**) 0.1 mM; (**E**) 1 mM; and (**F**) 10 mM.

HPSEC is an important technique used to reflect the distribution of molecular weight and protein aggregation. The HPSEC results of walnut protein samples are presented in Figure 3. The calibration curve of standard proteins measured under the same column is shown in Figure 3A. The HPSEC results of non-modified walnut protein show a polydisperse distribution with three major peaks, with retention times of 6.0 min (1.89% area, molecular weight distributed higher than 1000 kDa); 9.82 min (34.71% area, 282 kDa); and 11.77 min (63.41% area, 69 kDa) (Figure 3B). The continuous distribution of molecular weight indicates that walnut protein is made up of a series of different molecular weight proteins. The percentage area of the peak decreased as the concentration of MDA increased from 0 mM to 0.1 mM. Furthermore, a new peak of molecular weight at 50 kDa (64.18% area, molecular weight distributed from 20 to 50 kDa) appeared when the concentration of MDA was 0.1 mM. This may be due to the formation of low molecular weight polypeptides [29]. In contrast, when the concentration of MDA increased from 0.1 mM to 10 mM, the percentage area of molecular weight higher than 1000 kDa, 282 kDa, and 69 kDa steadily increased, and that of 50 kDa disappeared (Figure 3D–F). This indicates that proteins gradually aggregated at higher degrees of MDA-induced oxidation. These results imply that low MDA concentrations (≤1 mM) induced walnut protein to undergo small polypeptide chain scission, larger aggregates formed due to small chain aggregation [3], and crosslinking occurred as the MDA concentration reached 10 mM. These results are similar to that of soy protein oxidation induced by MDA [3].

### 3.5. Solubility of Proteins

Some of the functional properties of proteins used in the food industry, such as foaming, emulsification, and gel properties, are associated with solubility. A change in protein dissolution behavior can be used to evaluate the degree of protein denaturation. The solubility of native and oxidized walnut proteins is shown in Table 1. The native walnut protein was less soluble in water, only 11.78%, because the poorly soluble gluten makes up 70% of the protein complement [2]. The protein solubility declined (*p* < 0.05) with an increasing MDA concentration, and the solubility was decreased by 86% at 10 mM of MDA. Results of previous studies have shown that MDA-induced modification also reduces soy protein solubility [3]. Ye et al. [18] reported that the decrease in the solubility of peanut protein isolate induced by oxidation is due to the formation of insoluble protein oxidation aggregates. The MDA reacted with nucleophilic side chains groups of proteins to produce the Schiff base, which led to the covalent crosslinking of walnut protein and the formation of insoluble aggregates. This resulted in the eventual loss of protein solubility [30]. According to the HPSEC results of walnut protein samples, low MDA concentrations (1 mM) induced walnut protein to undergo small polypeptide chain scission, and larger aggregates formed due to small chain aggregation and crosslinking as the MDA concentration reached 10 mM, affecting the solubility of oxidized walnut protein. Furthermore, the intrinsic fluorescence results show that the tertiary structure of walnut protein was changed by MDA modification. Combined with the results of the changes in the carbonyl groups of oxidized proteins, it can be concluded that malondialdehyde oxidation causes the walnut protein to undergo some degree of denaturation, resulting in a decrease in solubility.

### 3.6. Foam Capacity (FC) and Foam Stability (FS)

**Figure 4:**
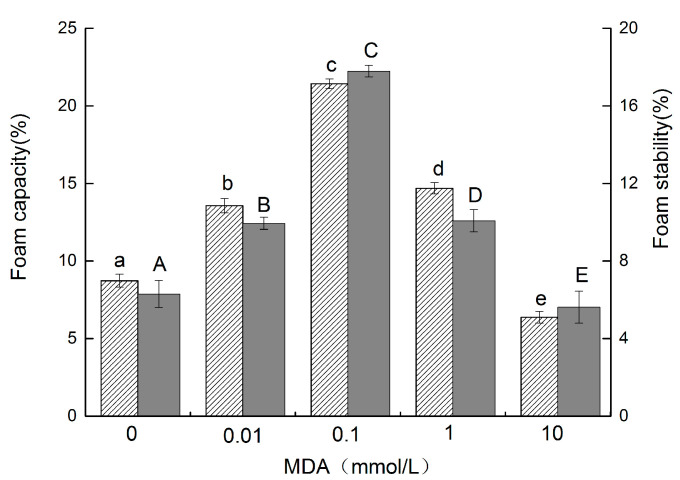
FC and FS of walnut protein at different concentrations of MDA (The FC is represented by the column with oblique lines 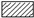, and the FS is represented by the gray column 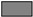. The different letters in the same indicator indicate significant difference at *p* < 0.05).

Foam is formed by gas dispersion in a continuous aqueous or liquid phase. Proteins in dispersions generate lower surface tension at the water–air interface, which helps to create foam and maintain its stability [31]. The FC and FS of the walnut protein samples are presented in Figure 4. The FC and FS of non-oxidized walnut protein were 8.73% and 6.30%, respectively. The FC and FS of modified walnut protein with an increasing concentration of MDA increased significantly (*p* < 0.05). The maximal FC and FS were obtained when walnut protein was oxidized by 0.1 mmol/L, to 21.43% and 17.80%, respectively. However, the FC and FS decreased to 6.37% and 5.62%, respectively, as the concentration of MDA increased to 10 mmol/L. These results are consistent with Wang’s study [32]. The increase in the net charge of the protein and interactions between hydrophobic residues caused by the unfolding of peptide chains led to lower surface tension [33] and higher protein flexibility, which allowed the protein to spread to the air–water interface more quickly. This was conducive to foam formation [34]. As the degree of oxidization was increased, the structure of walnut protein would become more unstable and extensive unfolding would disrupt the protein–lipid interactions at the interface, thus decreasing both FC and FS [31]. Foaming capacity could be affected by solubility, molecular flexibility, conformation, and protein molecular weight. Protein surface-hydrophobicity (H0) and molecular weight mainly influence the initial rate of protein adsorption at the air–water interface. A higher H0 and lower molecular weight help protein molecules more quickly diffuse to the air–water interface, which leads to encapsulating air particles and increasing FC. The protein solubility of walnut protein decreased (*p* 0.05) as the MDA concentration increased. However, our previous results showed that the surface hydrophobicity of walnut protein gradually increased with an increasing concentration of MDA oxidation. In addition, malondialdehyde oxidation can lead to partial covalent cross-linking of walnut protein molecules and degradation of protein peptide chains, resulting in the production of low molecular weight proteins.

### 3.7. Emulsifying Properties

**Figure 5:**
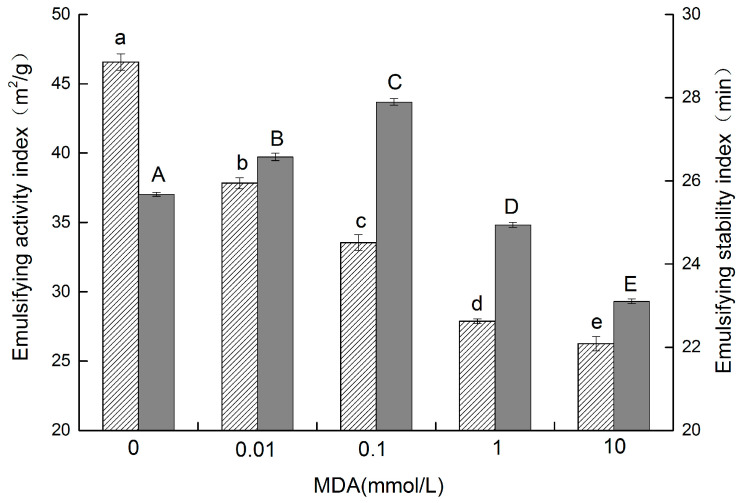
EAI and ESI of walnut protein at different concentrations of MDA (The EAI is represented by the column with oblique lines 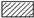, and the ESI is represented by the gray column 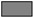. The different letters in the same indicator indicate significant difference at *p* < 0.05).

Emulsion is integral to a wide range of food properties. Since they possess both hydrophilic and oil-friendly groups, proteins can act as emulsifiers. The emulsifying properties of proteins are indicated by the emulsifying activity index (EAI) and the emulsification stability index (ESI). EAI refers to the ability of a protein to form an emulsion, and ESI refers to the ability of a protein to resist change in stability over a defined period of time [20]. The EAI of walnut protein (Figure 5) was reduced (*p* < 0.05) from 45.96 m^2^/g to 25.86 m^2^/g as the concentration of MDA increased from 0 mM to 10 mM. Studies have shown that good emulsion properties are related to better solubility and surface hydrophobicity [35]. Protein crosslinking and the formation of insoluble aggregates resulting from malondialdehyde oxidation decreased the ability of protein to absorb fat, reducing the EAI. This can be seen from the results of the molecular weight distribution (HPSEC) of oxidized walnut protein by MDA. A similar result was observed by Cao [36], who reported that Epigallocatechin-3-gallate at higher levels decreases the EAI of porcine myofibrillar protein.

As shown in Figure 5, compared to the change in EAI, the ESI of walnut protein increased from 25.67 min to 27.90 min as the concentration of MDA increased from 0 to 0.1 mM. Contrarily, the ESI of walnut protein decreased to 23.10 min when the concentration of MDA increased to 10 mM. These results are in agreement with a recent study that reported that low-concentration H_2_O_2_ oxidation increases the ESI of soy protein isolate, and that higher levels of H_2_O_2_ significantly interfere with the ESI [37]. These results indicate that the addition of MDA can improve the emulsifying activity of walnut protein. The partial unfolding of the protein structure caused by low concentrations of MDA (≤0.1 mM) exposed more hydrophobic groups hidden in the protein molecules and increased molecular flexibility. These changes were conducive to the formation of protein aggregates and adherence of the protein to the oil droplet surface. However, moderate oxidation increased the electrostatic repulsion between the droplets and the space resistance on the adsorption film [37], so the stability of protein emulsification was increased. However, the formation of insoluble aggregates and the decrease in surface hydrophobicity resulting from higher MDA-induced oxidation broke the adsorption film and protein molecular flexibility, thus, resulting in the decrease in protein emulsification stability. This result can also be informed by circular dichroism (CD) spectra measurement studies of oxidized walnut proteins. The oxidation of MDA caused the walnut protein to lose its ordered secondary structure, specifically the α-helix and β-sheet, and the random coil content also increased.

## 4. Conclusions

The effects of protein oxidization induced by MDA, which was selected as the representative of lipid peroxidation products, on the structure and functional properties of walnut protein were investigated, with MDA concentrations of 0, 0.01, 0.1, 1, and 10 mM. Compared with non-oxidized walnut protein, walnut protein modified by MDA-induced oxidation exhibited a dramatic change in structure and functionality. The findings show that MDA-induced oxidation altered the functional properties of walnut protein by disrupting its structure. MDA reacted with ε-amino groups and sulfhydryl groups to form protein aggregates, thus, facilitating protein carbonylation and the degradation of protein sulfhydryl groups, which are markers of protein oxidation. Furthermore, HPSEC also revealed protein degradation. Remarkably, decreases in α -helical and β-sheet content and decreases in fluorescence quenching revealed molecular rearrangement and unfolding of the tertiary structure. In addition, the data show that MDA-induced oxidation decreased solubility and had a negative effect on emulsifying activity. In parallel, low concentrations of MDA (≤0.1 mM) increased the emulsification stability and foaming properties (including FC and FS), but higher MDA concentrations (>0.1 mM) were detrimental to emulsification and foaming properties. This study discovered a link between MDA, a byproduct of lipid oxidation, and walnut protein oxidation, providing a foundation for quality control of walnut protein products during processing and storage. In particular, moderate oxidation induced by MDA (0.1 mM) can improve the functional properties of walnut proteins.

## Figures and Tables

**Table 1:** Protein carbonyl, free sulfhydryl, total sulfhydryl, and solubility of walnut protein incubated with different concentrations of MDA.

MDA	Protein Carbonyl	Free Sulfydryl	Total Sulfydryl	Solubility
(mM)	(nmole/mg)	(nmole/mg)	(nmole/mg)	(%)
0	3.12 ± 0.11 a	34.84 ± 0.90 a	167.49 ± 3.35 a	11.78 ± 0.14 a
0.01	4.64 ± 0.14 b	32.17 ± 0.91 a	148.06 ± 3.69 b	9.51 ± 0.24 b
0.1	5.26 ± 0.15 c	23.02 ± 1.23 b	126.73 ± 1.95 c	6.73 ± 0.21 c
1	9.09 ±0.17 d	20.79 ± 0.53 c	110.61 ± 1.73 d	4.51 ± 0.44 d
10	20.61 ± 0.15 e	9.85 ± 1.62 d	83.12 ± 4.04 e	3.50 ± 0.39 e

All values were measured in triplicate and expressed by the average of duplicate results ± standard errors. The different letters in the same indicator indicate a significant difference at the *p* < 0.05 level.

## Data Availability

The data presented in this study are available on request from the corresponding author.
